# Remote Ischemic Postconditioning: Harnessing Endogenous Protection in a Murine Model of Vascular Cognitive Impairment

**DOI:** 10.1007/s12975-014-0374-6

**Published:** 2014-10-29

**Authors:** Mohammad Badruzzaman Khan, Md Nasrul Hoda, Kumar Vaibhav, Shailendra Giri, Philip Wang, Jennifer L. Waller, Adviye Ergul, Krishnan M. Dhandapani, Susan C. Fagan, David C. Hess

**Affiliations:** 1 Departments of Neurology, Georgia Regents University, 1120 15th St, CA 1014, Augusta, GA 30912 USA; 2 Departments of Medical Laboratory, Imaging and Radiologic Sciences, Georgia Regents University, Augusta, GA USA; 3 Departments of Psychiatry, Georgia Regents University, Augusta, GA USA; 4 Departments of Biostatistics and Epidemiology, Georgia Regents University, Augusta, GA USA; 5 Departments of Physiology, Georgia Regents University, Augusta, GA USA; 6 Department of Neurosurgery, Georgia Regents University, Augusta, GA USA; 7 Program in Clinical and Experimental Therapeutics, College of Pharmacy, University of Georgia, Augusta, GA USA; 8 Charlie Norwood VA Medical Center, Augusta, GA USA; 9 Department of Neurology, Henry Ford Health Systems, Detroit, MI USA

**Keywords:** Vascular cognitive impairment, Remote ischemic postconditioning, Chronic cerebral hypoperfusion, Arterial stenosis, Transient ischemic attacks, White matter lesion

## Abstract

We previously reported that remote limb ischemic conditioning (RLIC; PERconditioning) during acute stroke confers neuroprotection, possibly due to increased cerebral blood flow (CBF). Vascular cognitive impairment (VCI) is a growing threat to public health without any known treatment. The bilateral common carotid artery stenosis (BCAS) mouse model is regarded as the most valid model for VCI. We hypothesized that RLIC (postconditioning; RIPostC) will augment CBF during chronic cerebral hypoperfusion (CCH) and prevent cognitive impairment in the BCAS model. BCAS using customized microcoil was performed in C57/B6 male mice to establish CCH. A week after the BCAS surgery, mice were treated with RIPostC-therapy once daily for 2 weeks. CBF was measured with laser speckle contrast imager at different time points. Cognitive testing was performed at 4-week post-BCAS, and brain tissue was harvested for biochemistry. BCAS led to chronic hypoperfusion resulting into impaired cognitive function as tested by novel object recognition (NOR). Histological examinations revealed that BCAS triggered inflammatory responses and caused frequent vacuolization and cell death. BCAS also increased the generation and accumulation of amyloid beta protein (Aβ), resulting into the loss of white matter (WM) and myelin basic protein (MBP). RIPostC-therapy showed both acute increase as well as sustained improvement in CBF even after the cessation of therapy for a week. RIPostC improved cognitive function, inhibited inflammatory responses, prevented the cell death, reduced the generation and accumulation of Aβ, and protected WM integrity. RIPostC is effective in the BCAS model and could be an attractive low-cost conventional therapy for aged individuals with VCI. The mechanisms by which RIPostC improves CBF and attenuates tissue damage need to be investigated in the future.

**Electronic supplementary material:**

The online version of this article (doi:10.1007/s12975-014-0374-6) contains supplementary material, which is available to authorized users.

## Introduction

The prevalence of dementia is expected to triple by 2050 [1]. In the past few decades, there has been an “Alzheimerization” of dementia with a tendency to attribute all cognitive decline to Alzheimer’s disease (AD) [2]. This view is now being revised with the recognition of the major vascular contribution to dementia [1, 3–5]. Vascular cognitive impairment (VCI) is the term that encompasses the clinical spectrum from mild cognitive dysfunction to vascular dementia. The NINDS Stroke Progress Review Group in 2012 cited “prevention of VCI” as a major research priority (http://www.ninds.nih.gov/find_people/ninds/OSPP/Stroke-Research-Priorities-Meeting-2012.htm).

The pathological hallmark of VCI is white matter (WM) damage from ischemia in the periventricular regions and centrum semiovale [1, 5]. The imaging correlate of this WM damage is “leukoaraiosis” [6]. The degree and severity of leukoaraiosis are associated with cognitive impairment, depression, gait abnormalities, and disability [6]. There is no known treatment once symptoms appear although observational studies suggest exercise (probably due to upregulation of endogenous protection) may slow down cognitive decline [7]. WM changes are mediated by vascular dysfunction and inflammation, blood-brain barrier (BBB) leakage, glial activation and injury to oligodendrocytes, and finally demyelination [1]. Reduction in the cerebral blood flow (CBF) leading to hypoperfusion is an early and characteristic finding [1].

Remote limb ischemic conditioning (RLIC) is the simple, inexpensive, and safe use of repetitive inflation-deflation procedure of a blood pressure (BP) cuff on the arm or leg to protect distant organs such as the brain, heart, and kidney from ischemic injury [8]. A number of preclinical studies also demonstrated that RLIC is effective at reducing injury in focal cerebral ischemia models (recently reviewed by us) [8]. We demonstrated that RLIC is protective and increases CBF in a murine model of thromboembolic stroke [9, 10]. Moreover, in patients with intracranial arterial stenosis, RLIC increased CBF as measured by SPECT [11]. Therefore, the mechanism of this protection may partially rely on the improvement of CBF.

There are a number of proposed animal models for VCI. In a recent review of all mouse models for VCI and vascular dementia [12], Bink and colleagues found that the mouse bilateral common carotid artery stenosis (BCAS) model to be the best and most valid [13]. This model reproduces cerebral hypoperfusion, inflammation, BBB damage, WM damage, and cognitive deficits of the human condition [13, 14]. It also avoids damage to the visual pathways, a complication of rat model of carotid ligation. We hypothesized that remote ischemic postconditioning (RIPostC) will improve CBF and behavioral outcomes and reduce inflammation and WM damage in this murine BCAS model of VCI.

## Materials and Methods

### Animals and Experimental Groups

The Institutional Animal Care and Use Committee of Georgia Regents University (GRU) approved the experimental procedures as per the National Institute of Health guidelines. C57BL/6 J wild-type male mice (10 ± 1 weeks old) were procured from Jackson Laboratory (Bar Harbor, Maine) and were housed in GRU’s AAALAC accredited facility. In these experiments, we use the term “remote ischemic postconditioning” (RIPostC) as we start the conditioning 1 week after the VCI procedure for 2-week period. We randomized 20 mice into 3 groups: a sham-operated group for procedures of BCAS and therapy (Sham group, *N* = 5), an untreated BCAS/VCI-induced group (with RIPostC sham procedure), i.e., BCAS group (*N* = 7), and a VCI-induced RIPostC-treated BCAS + RPostC-group (*N* = 8). The outcome measures were blinded. Cognitive function and CBF changes were considered as the primary outcomes to calculate the sample size of 5 in each group to provide >80 % power at α = 0.05, as reported by us earlier [10].

### Experimental Methods

Please see the Online Supplementary Information for the descriptive methodology. BCAS surgery and laser speckle contrast imaging (LSCI) were performed in mice sedated with buprenorphine and under isofluorane anesthesia. To produce BCAS, customized microcoils specially designed to produce VCI in the mouse were twined by rotating it around both common carotid arteries, as reported elsewhere [13]. Bilateral noninvasive RIPostC therapy or related sham procedure using a programmable cuff was performed daily for 2-weeks as reported earlier by us [9]. High-resolution LSCI (PSI system, Perimed Inc.) was used to image cerebral perfusion at different time points as indicated in the figure. Behavioral assessment by novel object recognition (NOR) test was performed as reported earlier by us [15]. The capability of the mouse to discriminate between a familiar versus novel object was determined as the discrimination index, *DI = (T*_*n*_ *-T*_*f*_ *)/(T*_*n*_ *+ T*_*f*_ *)*, where *T*_*n*_ is the time spent by the mouse with the novel object and *T*_*f*_ indicates the time spent with the familiar object.

At day 28, brain tissues were harvested after perfusion sacrifice, and the two hemispheres were immediately separated. One of the hemispheres was fixed in the chilled buffered formalin (10 %) for neuropathology and immunostaining, while the other one was snap frozen in liquid nitrogen for tissue biochemistry. Hematoxylin and eosin (HNE) staining was performed to estimate cell death and score the severity of pathology as reported by us [16]. Luxol fast blue (LFB)-neutral red staining was performed to detect the severity of WM lesion and fiber density [13]. Immunostaining for myelin basic protein (MBP) and Aβ_42_ were performed by using anti-MBP and anti-Aβ antibodies (Santa Cruz Biotechnology, USA), respectively. ELISA assay for Aβ_42_ in the brain tissue was performed using Aβ_42_ selective commercial kit and following the manufacturer’s protocol (AnaSpec, USA). Real-time quantitative PCR was performed as reported by us [17].

### Statistical Analysis

CBF was compared using repeated measure ANOVA only between the BCAS and BCAS + RIPostC therapy groups because the Sham group did not undergo any occlusive procedure, which can affect CBF. Other data were compared between all three groups using ANOVA. Wherever mentioned, Means with different letters are significantly different while “ns” stands for “no significant” difference (*p* < 0.05).

## Results

### RIPostC Therapy After BCAS Increases Cerebral Perfusion and Behavioral Outcome

**Fig. 1:**
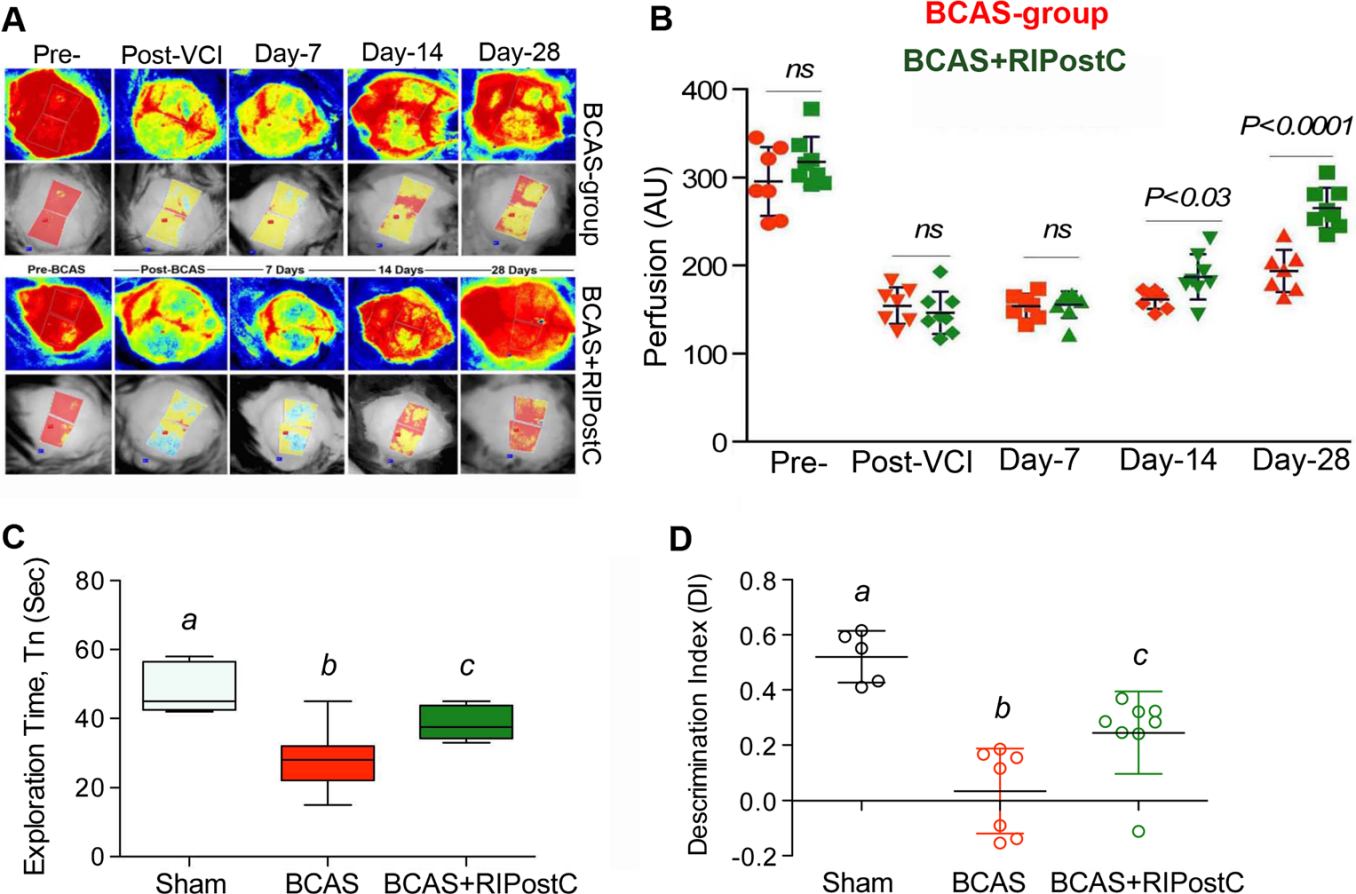
Detection of cerebral blood flow changes by laser speckle contrast imager (LSCI) and nonspatial working memory by novel object (NOR) recognition. **a** Representative contrast and photo overlay images of cerebral perfusion, **b** absolute value of cerebral perfusion in perfusion unit (PU) where “*red*” *symbols* indicate BCAS group while “*green*” *symbols* are for BCAS + RIPostC group, and **c** time of exploration (*T*_*n*_) spent with the novel object, and **d** the discrimination index (*DI*). Data presented as mean ± SD. *Pairs of means with different letters* are significantly different, *p* < 0.05

Figure 1a, b shows that there was no significant difference between the CBF of BCAS and BCAS + RIPostC groups at baseline before (295 ± 39 vs 317 ± 28 perfusion unit, PU) and immediately after (154 ± 20 vs 146 ± 23 PU) the BCAS surgery. Moreover, in agreement with the previous report [13], there was a slight but insignificant spontaneous improvement in the CBF of all BCAS groups with time and over days. However, there was no significant difference between the CBF of the two groups on day 7 post-BCAS and before initiating the RIPostC therapy (153 ± 14 vs 155 ± 15 PU). In comparison to untreated BCAS group, there was a significant increase (*p* = 0.03) in CBF after 7 days of RIPostC therapy in the BCAS + RIPostC group when measured the same day (day 14 post-BCAS) and 1 h after the treatment (161 ± 10 vs 192 ± 20 PU). We further continued the RIPostC therapy for 1 week more up to day 21 post-BCAS, and then discontinued it for the next 1 week. When measured at day 28, we again found significantly (*p* = 0.0001) increased CBF in the BCAS + RIPostC group as compared to the BCAS group (193 ± 23 vs 265 ± 23 PU). This demonstrates a sustained effect on the CBF even after the cessation of RIPostC therapy. We performed NOR test, which reliably evaluates the nonspatial working memory of the frontal subcortical region [18]. The results (Fig. 1c, d) show that the Sham group is more attracted toward a novel object, a characteristic exploratory feature in rodents. On the other hand, the BCAS group has lesser curiosity toward the exploration of the novel object as evident from the significantly decreased *T*_*n*_ (*p* = 0.0004) as well as *DI* (*p* < 0.0001) in comparison to the Sham group. This indicates that BCAS causes impairment of discriminative ability of the mouse toward novelty of an object. RIPostC therapy in the BCAS + RIPostC group significantly restored the *T*_*n*_ (*p* = 0.0336) and *DI* (*p* = 0.0256) scores as compared to the BCAS group. In conclusion, the data demonstrate that BCAS results in the loss of working memory while RIPostC attenuates this deficit.

### RIPostC Therapy After BCAS Attenuates Inflammatory Responses and Aβ_42_ Accumulation in the Brain

**Fig. 2:**
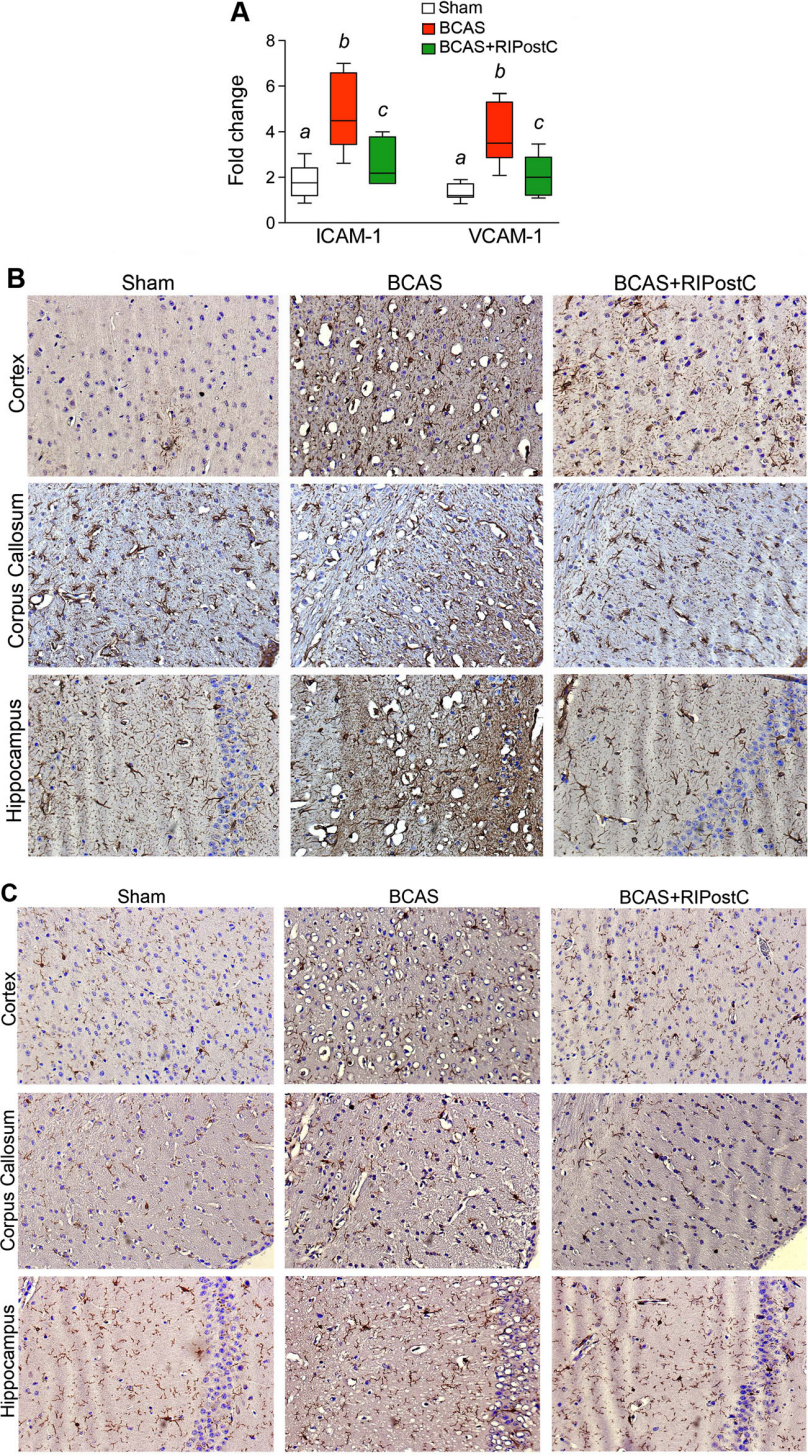
BCAS triggers vascular and glial inflammatory responses, which is attenuated by RIPostC. **a** Quantitation of mRNA expression of ICAM-1 and VCAM-1 as detected by the semiquantitative real-time PCR and normalized to beta-actin as housekeeping gene. Data are presented as mean ± SD. *Pairs of means with different letters* are significantly different, *p* < 0.05. **b**, **c** Representative immunostaining images from different regions of the brain (frontal cortex, corpus callosum (CC) and hippocampal CA1 region) as mentioned in the figure legend, showing activated glial response as detected by anti- GFAP and IBA1 antibodies, respectively (20×)

Chronic cerebral hypoperfusion triggers inflammatory responses, which impair microvascular function, increase generation of Aβ and decrease its clearance, and cause neurodegeneration deeper into the WM [1, 19–21]. We found that the gene expression of vascular inflammatory responses (ICAM-1 and VCAM-1) are significantly upregulated in the BCAS group as compared to Sham (Fig. 2). This subsequently led to glial activation and inflammation as evident by the increased GFAP staining and IBA-1 expression (Fig. 2). When treated with RIPostC therapy, the vascular inflammatory responses were significantly attenuated, and the glial activation was downregulated.

**Fig. 3:**
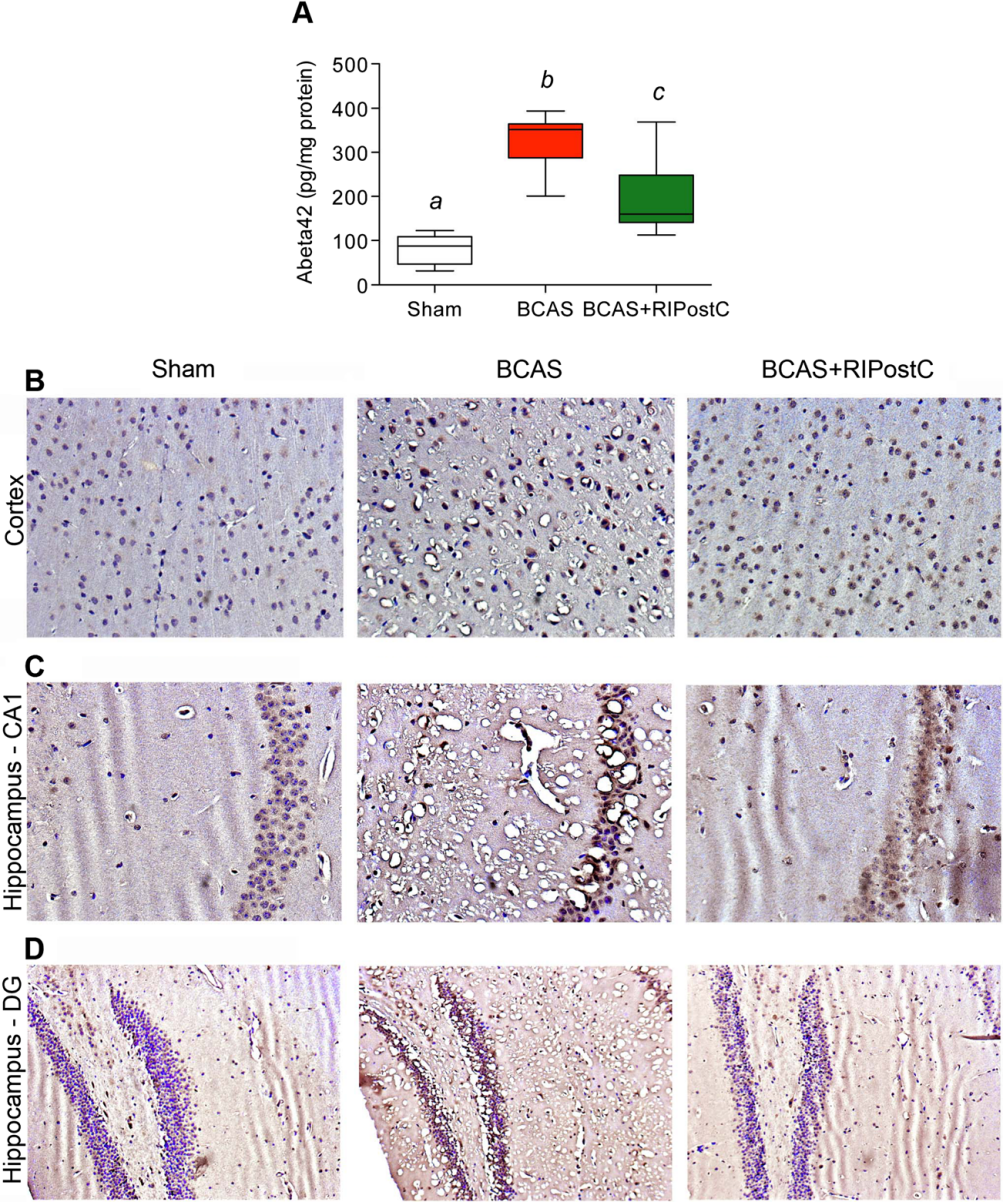
Progression of Aβ-type pathology in the brain 28-days after BCAS and its attenuation by RIPostC. Data are presented as mean ± SD*. Pairs of means with different letters* are significantly different, *p* < 0.05. **a** Quantification of Aβ_42_ by a commercial ELISA kit for mouse, and **b**, **d** representative images showing expression of Aβ in the different regions of the brain (frontal cortex and hippocampal CA1 as well as dentate gyrus (DG) regions) as detected by anti-Aβ antibody (20×)

Increased Aβ generation and resultant accumulation are one of the major characteristics of dementia and progression of related AD type pathology [21]. We quantified the Aβ level in the brain tissue by ELISA and investigated the accumulation pattern. Figure 3a shows that BCAS in 28 days led to significant generation and accumulation of Aβ_42_ (4-fold increase) in the brain of BCAS group as compared to the Sham group (*p* < 0.0001). RIPostC therapy for 2 weeks decreased the Aβ_42_ content (2-fold decrease) in BCAS + RIPostC-group as compared to BCAS group (*p* = 0.0051). Since Aβ plaque is a hallmark of dementia and AD-type pathology, we next investigated the expression and accumulation pattern of Aβ in the different regions of the brain. At 28-day post-BCAS, we did not find major plaque formation, but the expression of Aβ was increased after BCAS in every region of the brain (Fig. 3b–d). The representative images from the frontal cortex (an area associated with working memory) and the hippocampus show that at day 28, Aβ started accumulating around the neuronal cell body in the BCAS group, which was reduced in the BCAS + RIPostC group.

### RIPostC Therapy After BCAS Prevents Cell Death and Demyelination

**Fig. 4:**
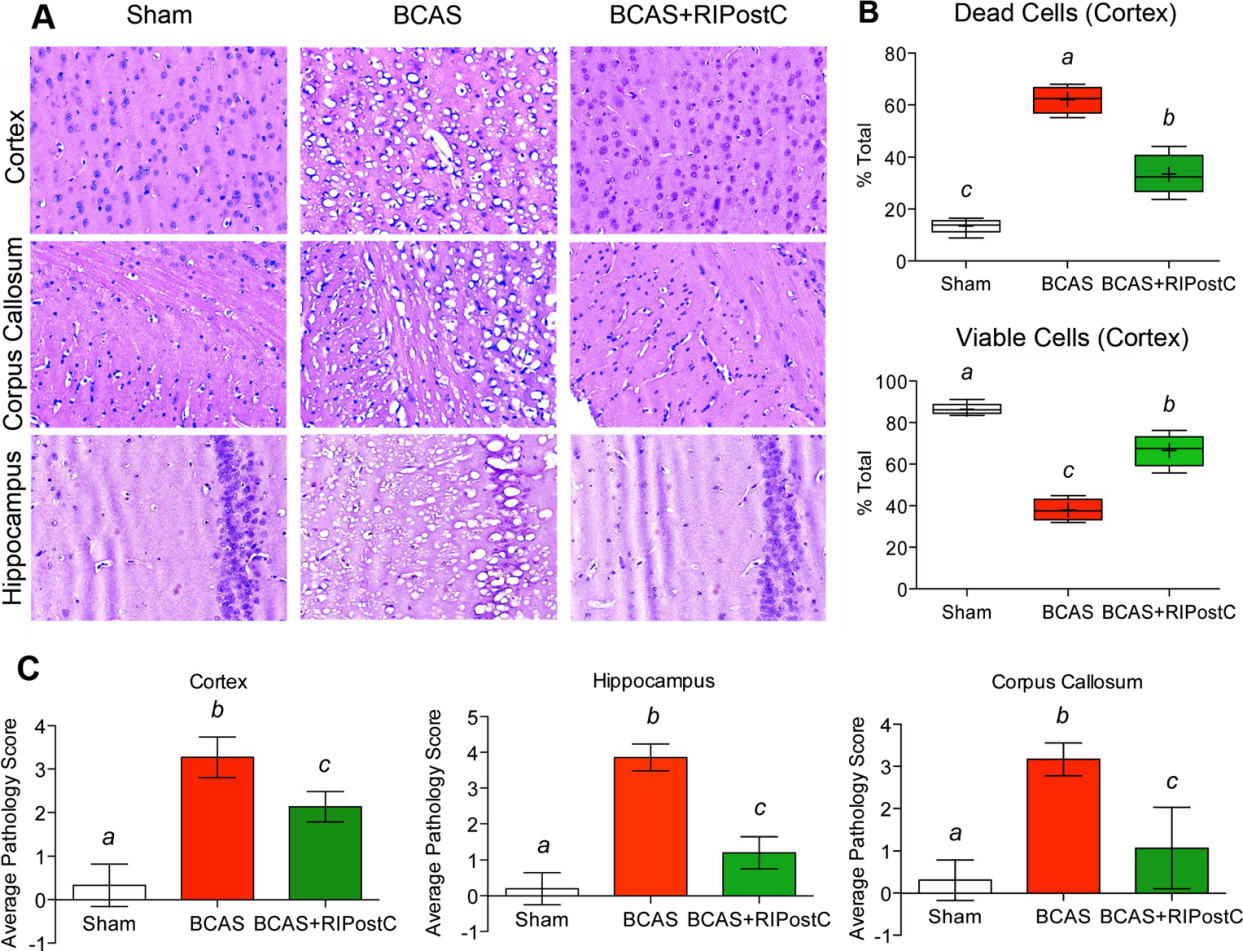
Histopathological changes in the brain due to BCAS and its modulation by RIPostC therapy as detected by the hematoxylin-eosin (HNE) staining. **a** Representative HNE images from different regions of the brain (frontal cortex, corpus callosum (CC), and hippocampal CA1 region) as mentioned in panels (20×), showing significant number of vacuoles and pyknotic dead cells after BCAS, and its protection by RIPostC, **b** quantitation of dead and viable cells at ×10 magnification in the cortical region, and **c** the average severity score of pathology in the above regions

HNE staining showed frequent vacuolization and pyknotic dead cells both in the cortical and WM regions after BCAS with moderate to severe neuropathological scores in different regions (Fig. 4) [16, 22]. When counted in the identical cortical region, % cell death was significantly increased while % viable cell was decreased in the BCAS group at day 28 as compared to the Sham. RIPostC therapy for 2 weeks robustly prevented the cell death and vacuolization in the BCAS + RIPostC group as compared to the BCAS group. RIPostC therapy also reduced the average severity score of pathology in the brain due to BCAS [16].

**Fig. 5:**
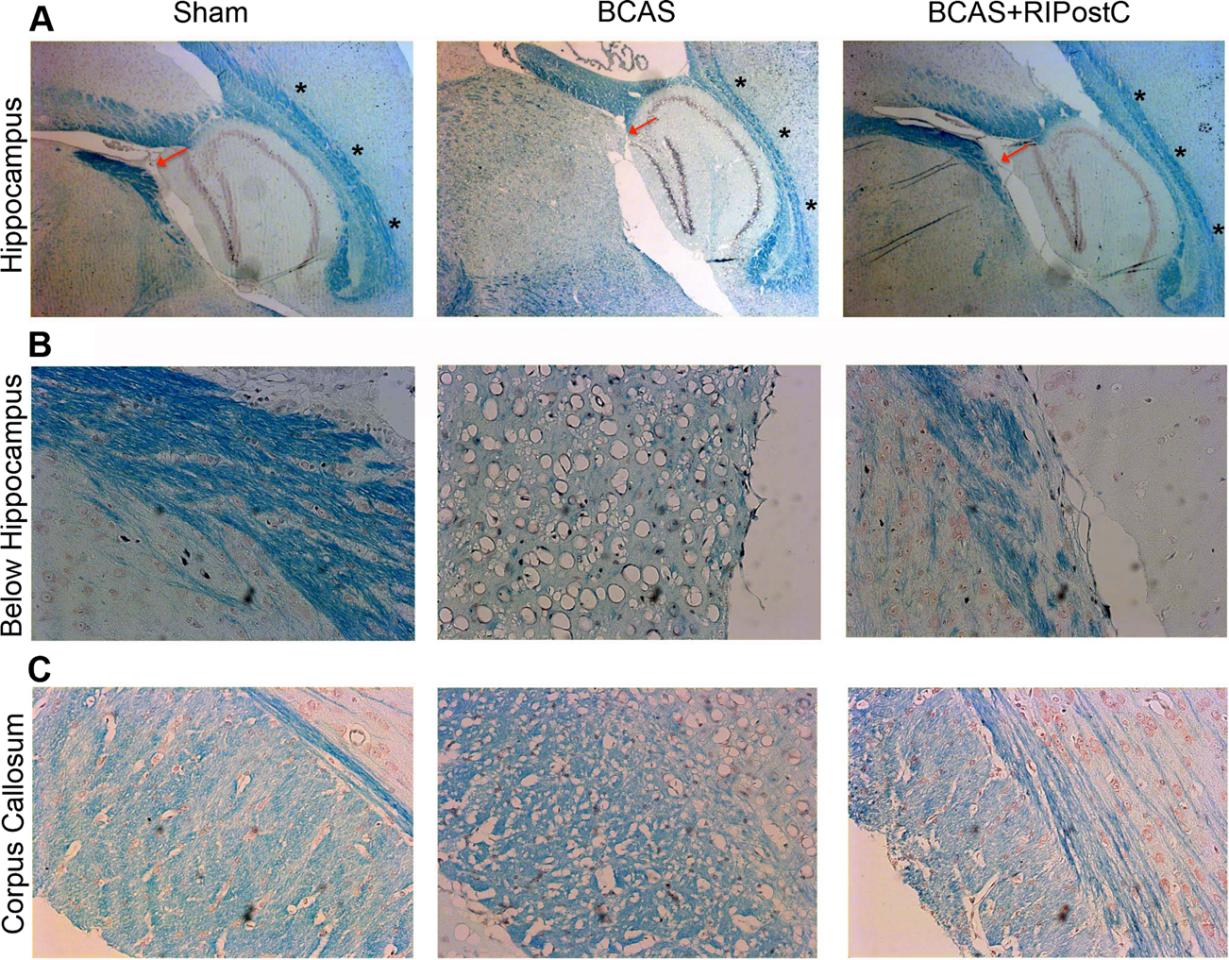
Luxol fast blue (LFB) staining for the detection of white matter (WM) changes after BCAS and its preservation by RIPostC therapy in the regions nearby hippocampus and corpus callosum. **a** Representative images (2.5×) from sagittal sections of hippocampus showing shrinkage above the CA1 region (as indicated by *black asterisks*) after BCAS. **b** Corresponding magnified images (×20) of the region below the hippocampus as indicated by *red arrow in each panel*, showing significant loss in the integrity of WM after BCAS and its augmentation by RIPostC. **c** Representative LFB images from the corpus callosum region showing significant degeneration by BCAS and its protection by RIPostC which can be more severe during long-term follow-up

**Fig. 6:**
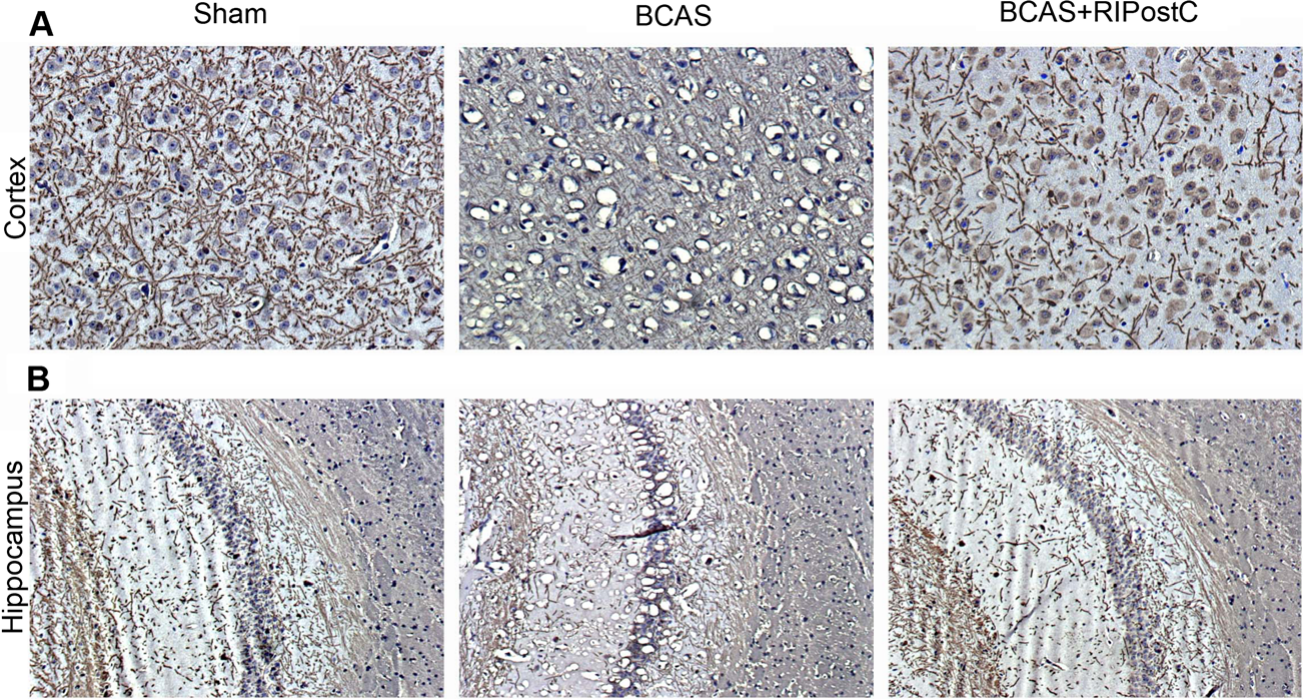
Loss in myelin basic protein (MBP) after BCAS and its protection by RIPostC therapy. **a** Representative images (10×) from the frontal cortical region showing significant vacuolization, and loss in MBP after BCAS as detected by anti-MBP immunostaining. **b** Representative images from the area showing CA1 region of hippocampus (10×)

We next investigated the integrity of WM and changes in the MBP expression in the different regions of the brain. Representative images (Fig. 5) show severe WM degeneration, and axonal loss as indicated by changes in LFB staining occurred after the BCAS in the different regions of the untreated BCAS group. At 28-day post-BCAS, such changes were present in the corpus callosum and hippocampal regions. RIPostC therapy robustly prevented the WM degeneration. Similarly, there was a severe loss in MBP expression in the frontal cortical region of the untreated BCAS group, which was preserved after RIPostC therapy in the BCAS + RIPostC group (Fig. 6). Similar to the LFB staining, changes in the MBP expression were more prominent in the cortical region compared to other regions in a 28-day survival period.

## Discussion

This is the first report in any animal model of VCI where RLIC has been tested. Our major findings are that RIPostC in the murine BCAS model improves CBF and cognitive function and reduces inflammation and neurodegeneration. Long-term (2 weeks) RIPostC therapy significantly increased the CBF in a sustained fashion and improved cognitive function. CBF remain increased for at least 1 week even after the cessation of long-term RIPostC therapy (Fig. 1). We also found reduced Aβ level in RIPostC-treated group that might be attenuated due to the improved CBF and subsequent clearance of Aβ [19]. BCAS in mice triggers a proinflammatory milieu and impairs microvascular dysfunction, as evident by the increased gene expression of ICAM-1 and VCAM-1 (Fig. 2) [20]. ICAM-1 and VCAM-1 promote adhesion phenomena resulting into disintegration of BBB and increased infiltration of proinflammatory immune cells, which amplifies the neuro-glial inflammation, WM degeneration, and cell death (Figs. 2, 3, 4, 5, 6). When treated, RIPostC therapy not only reduced the vascular inflammatory responses but it also downregulated GFAP (astrocytes) and IBA-1 (microglial) expressions, subsequently leading to reduced WM changes and neurodegeneration.

There are no known treatments for VCI. Therapies are needed to prevent the transition and progression of the disease process to dementia. A potential target population for RLIC may be patients with “leukoaraiosis” on MRI. The Leukoaraiosis and Disability cohort (LADIS) is a European multicenter collaboration with the aim to predict disability in the patient aged 65–84 years with leukoaraiosis [6, 22]. These patients suffer from cognitive impairment, gait instability and falls, depression, and urinary incontinence. The degree of leukoaraiosis is associated with cognitive impairment, and the progression of leukoaraiosis on MRI strongly predicts cognitive decline [23, 24]. There is no known treatment to slow down the cognitive decline, but observational studies suggest that physical activity (which modulates endogenous protection as well as CBF similar to RLIC) might be of benefit [7]. From the LADIS cohort, estimates of sample size for a clinical trial with an intervention to reduce progression of WM changes on MRI range from 58 to 70, highlighting the potential translational pathway for RLIC. Moreover, RLIC is feasible for long-term treatment and comparatively more conventional for aged individuals with gait problem. A small size Chinese trial reported that RLIC increases CBF in patients suffering from intracranial arterial stenosis and prevents recurrent stroke [11]. It also demonstrates that this therapy using a BP cuff has long-term feasibility, as they were able to treat patients for 300 days.

Our study has several limitations. First, our number of animals is small (*N* = 20), but the study is randomized, blinded, and adequately powered. The effects observed were robust, however, indicating that large sample sizes are not needed. Second, while the BCAS model is regarded as the most valid for VCI, it does not recapitulate small vessel disease, the underlying pathophysiology of VCI. While the model produces chronic hypoperfusion, it does this via obstruction of the larger blood vessels affecting microvascular flow but the pathology is itself not intrinsic in the smaller vessels. Moreover, the disease process has a defined start whereas in humans, it is insidious with slow onset. Third, we treated and followed these mice for a relatively shorter period of time, 28 days. Fourth, we used young male mice, and VCI is a disease of the elderly. Fifth, in this translation report, we did not investigate the mechanism of protection by RIPostC. We now have ongoing work to test RIPostC therapy in aged mice of both sexes; MRI to see WM changes, and study the possible mechanism of vascular protection by long-term RIPostC therapy.

In summary, RIPostC therapy increases CBF and improves cognitive performance in a murine model of VCI. This therapy is highly translatable to humans. If successful, this no-additional cost therapy using BP cuff will be an “exercise equivalent” which will be highly convenient for elderly patients as well as caretakers to perform, and therefore, may change the current therapeutic paradigm of VCI in humans.

## Electronic supplementary material

Below is the link to the electronic supplementary material.

ESM 1 (DOC 75 kb)
