# Supplementary material for: Remote Ischemic Postconditioning: Harnessing Endogenous Protection in a Murine Model of Vascular Cognitive Impairment
Source: Transl Stroke Res. 2014 Oct 29;6(1):69–77. doi: 10.1007/s12975-014-0374-6 (PMC4297613; doi:10.1007/s12975-014-0374-6)
Supplement: Supplementary file 1 — (DOC 75 kb) [file 12975_2014_374_MOESM1_ESM.doc]

**ONLINE SUPPLEMENTARY METHODS**

**Animal Model of Vascular Cognitive Impairment (VCI)**

Bilateral Common Carotid Artery Stenosis (BCAS) procedure in the mouse was performed as described elsewhere.1 Briefly, mice were subjected to BCAS and subsequent cerebral chronic hypoperfusion using microcoils specially designed for the mouse (microcoil specifications: piano wire diameter 0.08mm, internal diameter 0.18mm, coiling pitch 0.5mm, and total length 2.5mm; Sawane Spring Co Ltd, Japan). To induce VCI in the Buprenex sedated mice under isofluorane; a midline cervical skin incision was made ventrally. The two common carotid arteries (CCA) were exposed one-by-one, freed from their sheaths, and a microcoil was twined by rotating it around the each CCA, as published elsewhere. The site of surgery was closed, and mice were observed and taken care of post-surgery until conscious and recovered to freely access food and water *ad libitum*. In Sham group, the skin was incised and the two common carotid arteries were exposed one by one.

**Non-Invasive RIPostC Therapy**

Non-invasive RIPostC therapy using a programmable cuff or its sham procedure was performed as reported earlier by us,2 with certain modifications. Bilateral RIPostC-therapy was performed in both hind limbs simultaneously (4 cycles x 5 min duration of each cycle x 5 min interval between each cycle) for 2 weeks from day 7 to day 21 post-BCAS.

**CBF by Laser Speckle Contrast Imager (LSCI)**

High resolution LSCI (PSI system, Perimed Inc.) was used to image cerebral perfusion and record CBF before BCAS (baseline), immediately after BCAS, at day 7 after BCAS and before initiating RIPostC therapy. CBF was also measured at day 14 post-BCAS (that is 7 days after initiating daily RIPostC therapy), and lastly at day 28 after a 1-week interval from the time when the RIPostC therapy was ended on day 21 post-BCAS. As shown in Fig 1, regions of interest (ROIs) between the bregma and lambda in the two hemispheres were selected for over all perfusion in the area of two hemispheres. Briefly, whenever required the mouse was anesthetized using isofluorane, body temperature was maintained at 37 ±0.5 °C, and the skull was shaved and exposed by a midline skin incision. The skull was cleaned gently with sterile phosphate buffered saline (PBS) and using cotton applicator. Finally it was kept wet and non-toxic silicon oil was applied on the skull, which improves imaging. Perfusion images were acquired using PSI system with a 70 mW built-in laser diode for illumination and 1388 x 1038 pixels CCD camera installed 10 cm above the skull (speed 19 Hz, and exposure time 6 mSec). Acquired images were analyzed for changes in CBF (cerebral perfusion) using a dedicated PIMSoft program (Perimed Inc.).

### Assessment of Cognitive Function by Novel Object Recognition (NOR) Test

Behavioral assessment by NOR test was performed as reported earlier by one of us.3 Briefly, mice were habituated in an activity box and familiarized with two identical objects placed at a set distance apart. On day 28, the mouse was given similar trial and the time spent (*Tf*) with the familiar object was recorded. After the familiar object trial, the mouse was then removed from the environment for a set amount of time and one of the two previously used (familiar) objects was replaced with a novel object that was different from the familiar object in shape, texture, and appearance. The time spent (*Tn*) by the mouse with the novel object upon exposure during the probe trial was then recorded in one 5-min trial. This test is based on the natural tendency of mice to investigate a novel object rather than a familiar one, which reflects the use of learning and recognition memory processes. The capability of the mouse to discriminate between a familiar vs. novel object was determined as the discrimination index, *DI = (Tn-Tf)/(Tn+Tf)*.

**Sample Collection**

Mice were deeply anesthetized, and blood was collected via cardiac puncture followed by perfusion sacrifice with 50 mL chilled 1X PBS (pH 7.4). The brain was harvested and the two hemispheres were immediately separated. One of the hemispheres was fixed in the chilled buffered formalin (10%) for neuropathology and staining procedures, while the other one was snap frozen in liquid nitrogen for tissue biochemistry.

**Histology and Immunostaining**

To quantitate the cell death, sagittal sections (6-7 μm thick) were obtained from the paraffin embedded tissue blocks and stained with standardized procedure for hematoxylin and eosin (HNE). Non-viable neurons and were determined by the presence of hyper eosinophilic cytoplasm and pyknotic nuclei.4 The percentage of non-viable neurons was calculated for each brain region (average of 3 levels per region). The severity of neuropathology was scored as reported by us. 4 The investigator was blinded to groups.

Fiber density of luxol-fast-blue (LFB)-neutral red staining was performed to detect the severity of WM lesion.1, 5, 6 Briefly, de-waxed rehydrated sections were immersed in the LFB solution (Solvent Blue 38, Sigma) at 60 °C overnight. Excess stain was removed by 95% ethanol treatment followed by washing with deionized water. Grey and white matter differentiation was initiated with the treatment of 0.05% aqueous lithium carbonate (Sigma) for 30 second followed by 70% ethanol until the nuclei are decolorized. Sections were immersed in neutral red solution (Sigma) for 30 min and washed in deionized water. They were dehydrated in ethanol gradient (70 – 100%), and finally cleared in xylene and mounted with cytoseal.

For other immunostaining, sections after standard preparatory procedures and antigen retrieval were incubated with either anti-MBP (C-16 clone, SC-13914, Santa Cruz, CA, USA; 1:100 dilution) or an antibody against beta-amyloid (Aβ; H-43 clone, SC-9129; 1:100 dilution) overnight, followed by incubation with appropriate biotinylated secondary antibody (Vector Laboratories, Burlingame, CA; 1:200 dilution) for 1 hour. To visualize the immunoreactivity, sections were finally incubated with an avidin–biotin peroxidase complex solution (Vectastain ABC kit, Vector Laboratories, diluted 1:100) for 30 min, followed by diaminobenzidine (DAB) as the substrate (Vector Laboratories). Images were captured with Zeiss microscope integrated with Axio Vision software.

**ELISA for Beta-amyloid (1-42):**

Aβ42 assay in the brain tissue was performed using highly sensitive commercial kit for the quantitation of Aβ42 in the mouse and rat tissue (Cat # 55554, AnaSpec Inc., Fremont CA). Briefly, 100 mg of brain tissue was homogenized in a buffer pH 8.0 (5M Guanidine HCl, 50 mM Tris-HCl) and incubated at room temperature for 4 hrs with intermittent mixing. Samples were diluted into 1:40 ratio using dilution buffer, and the ELISA for Aβ42 was performed following manufacturer’s protocol (AnaSpec). Finally, absorbance (OD) was measured at 450 nm using a micro plate absorbance reader within 20 min after adding the stop solution. This kit has more sensitivity and negligible interference from Aβ40­.

**Isolation of RNA, synthesis of cDNA, and real-time PCR**

Real time quantitative PCR was performed as reported by Hoda et al.7 Briefly, the purified RNA was reverse-transcribed into complementary deoxyribonucleic acid (cDNA) using iScript reagents from Bio-Rad on a programmable thermal cycler (PCR-Sprint, Thermo Electron, Milford, MA). 50 ng of cDNA was amplified in each real-time PCR using a BioRad iCycler, ABgene reagents (Fisher Scientific, Pittsburgh, PA) and specific primers (Table 1). Beta-actin was used as the internal control for normalization.

**Supplementary References:**

1. Shibata M, Ohtani R, Ihara M, Tomimoto H. White matter lesions and glial activation in a novel mouse model of chronic cerebral hypoperfusion. *Stroke*. 2004;35:2598-2603

2. Hoda MN, Bhatia K, Hafez SS, Johnson MH, Siddiqui S, Ergul A, et al. Remote ischemic perconditioning is effective after embolic stroke in ovariectomized female mice. *Transl Stroke Res*. 2014

3. Wakade C, Sukumari-Ramesh S, Laird MD, Dhandapani KM, Vender JR. Delayed reduction in hippocampal postsynaptic density protein-95 expression temporally correlates with cognitive dysfunction following controlled cortical impact in mice. *Journal of neurosurgery*. 2010;113:1195-1201

4. Mangalam A, Poisson L, Nemutlu E, Datta I, Denic A, Dzeja P, et al. Profile of circulatory metabolites in a relapsing-remitting animal model of multiple sclerosis using global metabolomics. *Journal of clinical & cellular immunology*. 2013;4

5. Ihara M, Tomimoto H. Lessons from a mouse model characterizing features of vascular cognitive impairment with white matter changes. *Journal of aging research*. 2011;2011:978761

6. Kitaguchi H, Tomimoto H, Ihara M, Shibata M, Uemura K, Kalaria RN, et al. Chronic cerebral hypoperfusion accelerates amyloid beta deposition in appswind transgenic mice. *Brain research*. 2009;1294:202-210

7. Hoda MN, Singh I, Singh AK, Khan M. Reduction of lipoxidative load by secretory phospholipase a2 inhibition protects against neurovascular injury following experimental stroke in rat. *Journal of neuroinflammation*. 2009;6:21

## Table 1: Oligonucleotide primer sequence used in RT- PCR

| **Primers** | **Sequence 5'- 3'** |
| --- | --- |
| ICAM-1 Forward-  Reverse- | ACGCAGAGGACCTTAACAGTCTAC  GCTTCACACTTCACAGTTACTTGG |
| VCAM-1 Forward-  Reverse- | TGAACAGACAGGAGTTTTCTTCAG  ATATCCTCAATGACGGGAGTAAAG |
| Bet-Actin Forward-  Reverse- | GTTTGAGACCTTCAACACCCC  GTGGCCATCTCCTGCTCGAAGTC |
